# Demographic and Health Behavior Factors Associated With Clinical Trial Invitation and Participation in the United States

**DOI:** 10.1001/jamanetworkopen.2021.27792

**Published:** 2021-09-29

**Authors:** Courtney P. Williams, Nicole Senft Everson, Nonniekaye Shelburne, Wynne E. Norton

**Affiliations:** 1 Division of Cancer Control and Population Sciences, National Cancer Institute, Rockville, Maryland

## Abstract

**Importance:**

Representative enrollment in clinical trials is critical to ensure equitable and effective translation of research to practice, yet disparities in clinical trial enrollment persist.

**Objective:**

To examine person-level factors associated with invitation to and participation in clinical trials.

**Design, Setting, and Participants:**

This cross-sectional study analyzed responses from 3689 US adults who participated in the nationally representative Health Information National Trends Survey, collected February through June 2020 via mailed questionnaires.

**Exposures:**

Demographic, clinical, and health behavior–related characteristics.

**Main Outcomes and Measures:**

History of invitation to and participation in a clinical trial, primary information sources, trust in information sources, and motives for participation in clinical trials were described. Respondent characteristics are presented as absolute numbers and weighted percentages. Associations between respondent demographic, clinical, and health behavior–related characteristics and clinical trial invitation and participation were estimated using survey-weighted logistic regression models.

**Results:**

The median (IQR) age of the 3689 respondents was 48 (33-61) years, and most were non-Hispanic White individuals (2063 [59%]; non-Hispanic Black, 452 [10%]; Hispanic, 521 [14%]), had more than a high school degree (2656 [68%]), were employed (1809 [58%]), and had at least 1 medical condition (2535 [61%]). Overall, 439 respondents (9%) had been invited to participate in any clinical trial. Respondents with increased odds of invitation were non-Hispanic Black compared with non-Hispanic White (adjusted odds ratio [aOR], 1.85; 95% CI, 1.13-3.02), had greater than a high school education compared with less than high school education (eg, ≥college degree: aOR, 4.84; 95% CI, 1.89-12.39), were single compared with married or living as married (aOR, 1.68; 95% CI, 1.04-2.73), and had at least 1 medical condition compared to none (eg, 1 medical condition: aOR, 2.25; 95% CI, 1.32-3.82). Respondents residing in rural vs urban areas had 77% decreased odds of invitation to a clinical trial (aOR 0.33; 95% CI 0.17-0.65). Of invited respondents, 199 (47%) participated. Compared with non-Hispanic White respondents, non-Hispanic Black respondents had 72% decreased odds of clinical trial participation (aOR, 0.28; 95% CI, 0.09-0.87). Respondents most frequently reported “health care providers” as the first and most trusted source of clinical trial information (first source: 2297 [59%]; most trusted source: 2597 [70%]). The most frequently reported motives for clinical trials participation were “wanting to get better” (2294 [66%]) and the standard of care not being covered by insurance (1448 [41%]).

**Conclusions and Relevance:**

The findings of this study suggest that invitation to and participation in clinical trials may differ by person-level demographic and clinical characteristics. Strategies toward increasing trial invitation and participation rates across diverse patient populations warrant further research to ensure equitable translation of clinical benefits from research to practice.

## Key Points

**Question:**

What person-level factors are associated with US adults’ invitation to and participation in clinical trials?

**Findings:**

In this cross-sectional study of 3689 adults, 9% were invited to participate in a clinical trial, and of those, 47% participated. Respondents had higher odds of clinical trial invitation if they were non-Hispanic Black, college educated, single, or urban-dwelling or had medical conditions; non-Hispanic Black respondents had lower odds of clinical trial participation.

**Meaning:**

In this study, clinical trial invitation and participation differed by person-level demographic and clinical characteristics, reinforcing the need for strategies encouraging generalizable and equitable translation of research to practice.

## Teaser

This cross-sectional study examines demographic, clinical, and health behavior–related factors associated with invitation to and participation in clinical trials in the US.

## Introduction

Evidence-based evaluation of new health care treatments and interventions via clinical trials is critical for improving individual and population health. As of April 2021, ClinicalTrials.gov listed 291 087 ongoing trials, including 158 827 drug or biologic interventions and 96 449 behavioral interventions.^1,2^ However, clinical trial participants infrequently represent the real-world population in which the intervention will be applied.^3,4^ Several patient population subgroups remain underrepresented in trials, including older adults, people who belong to racial or ethnic minority groups, persons with multiple comorbid conditions, and individuals residing in rural locations.^5,6,7^ Disparities between trial participants and real-world populations are problematic, since results from these trials provide the evidence base for real-world clinical practice guidelines and public health services.^8^

Previous research has shown that person-level factors, such as sociodemographic characteristics, may affect whether individuals are invited to participate in clinical trials, while motivations and beliefs may affect decisions to participate if invited.^9,10,11,12,13,14^ Much of the existing literature on trials tends to be condition-specific, focused on participation rather than invitation, and specific to subpopulations of the US public. To build on the existing literature base, investigation into the larger US public’s recent invitation to, participation in, and motivations surrounding clinical trials is needed. This study seeks to understand person-level factors associated with invitation to and participation in clinical trials in a large, nationally representative sample of US adults. We also describe respondent primary trial information sources, trust in information sources, and motives for participation in clinical trials to inform future efforts addressing disparities in clinical trial invitation and participation.

## Methods

### Study Design and Sample

This cross-sectional study used data from the National Cancer Institute’s Health Information National Trends Survey (HINTS) 5 Cycle 4, collected February through June 2020 via mailed questionnaires. HINTS is a publicly available, cross-sectional, nationally representative survey of civilian, noninstitutionalized US adults that assesses knowledge of, attitudes toward, and use of health-related information.^15,16^ HINTS 5 Cycle 4 included 7 new questions regarding clinical trials (eFigure in the Supplement).^15^ Initial constructs and associated items were included based on published literature representing a range of clinical conditions,^9,17^ racial and ethnic groups,^17^ and nationalities.^17,18,19^ Two rounds of cognitive testing^20^ were conducted with a diverse sample of 30 individuals (15 per round). Results informed the refinement of items and response options for inclusion in the survey. A plain language definition of clinical trials with 2 examples was included to standardize respondent interpretation and to specify that trials include drug and behavioral interventions. HINTS data are deidentified and thus exempt from review by the US National Institutes of Health Office of Human Subjects Research Protections. Respondents provided consent to participate in HINTS. This study follows the Strengthening the Reporting of Observational Studies in Epidemiology (STROBE) reporting guidelines.^21^

### Primary Outcome: Clinical Trial Invitation and Participation

Invitation to a clinical trial was assessed with a single yes or no item, “Have you ever been invited to participate in a clinical trial?” If the respondent answered yes, they were asked the single yes or no item, “Did you participate in the clinical trial?”

### Secondary Outcomes: Information Sources, Trust, and Motives for Participation in Clinical Trials

Respondents were asked about their current knowledge of clinical trials, where they would first go to get information about a hypothetical trial, and who they would most trust as a source of information about a hypothetical trial. Respondents were also asked to rate factors (eg, helping others, payment for participation) that would influence their decision to participate in a hypothetical clinical trial on a 4-point scale (a lot, somewhat, a little, not at all). Influential factors in trial decision-making were dichotomized as a lot vs the combined scores of somewhat, a little, and not at all.^22^ Separated scores for all response options are shown in eTable 1 in the Supplement.

### Covariables: Respondent Characteristics

Self-reported demographic, clinical, and health behavior–related respondent characteristics were included from the HINTS survey data for descriptive comparison and multivariable analysis. Demographic variables included age, sex, race and ethnicity (race: White, Black or African American, American Indian or Alaska Native, Asian Indian, Chinese, Filipino, Japanese, Korean, Vietnamese, other Asian, Native Hawaiian, Guamanian or Chamorro, Samoan, other Pacific Islander; ethnicity: not of Hispanic, Latino/a, or Spanish origin; Mexican, Mexican American, Chicano/a; Puerto Rican; Cuban; another Hispanic, Latino/a, or Spanish origin), education, feelings about present income, marital status, rural or urban residence,^23^ Census region, employment status, and health insurance status. Clinical variables included number and type of diagnosed medical conditions and self-reported health. Health behavior–related variables included smoking status, hazardous drinking,^24^ weekly exercise, and annual health care visits.

### Statistical Analysis

All analyses were weighted to provide nationally representative estimates using complex survey methodology with jackknife replicate weights for accurate standard errors.^15^ Mean differences, or effect sizes, were calculated using unweighted Cohen *d* or Cramer *V* to determine the magnitude of associations in bivariate associations.^25^ Associations between respondent demographic, clinical, and health behavior–related characteristics and clinical trial invitation and participation were estimated using adjusted odds ratios (aOR) and 95% CIs from exploratory survey-weighted logistic regression models. All covariables were included in the model exploring clinical trial invitation. Due to reduced sample size limiting power to determine magnitude of bivariate associations in the model estimating clinical trial participation, covariables were restricted based on past research demonstrating differential rates of clinical trial participation associated with age, race and ethnicity, sex, education, income, urban or rural residence, health insurance status, and health status.^26,27,28,29,30^ Covariable multicollinearity was examined using the variance inflation factor. Sensitivity analyses were conducted excluding respondents who reported knowing nothing about clinical trials. Trial participation motives, information seeking, and trust were described by (1) the overall sample, (2) respondents who participated in a clinical trial, and (3) respondents who were invited but did not participate in a clinical trial. All analyses were performed using SAS version 9.4 (SAS Institute, Cary, NC).

## Results

### Sample Characteristics

A total of 15 347 HINTS 5 Cycle 4 surveys were mailed. Of mailed surveys, 3977 were returned, 3865 were eligible for analysis (weighted response rate, 37%), and 3689, with complete information on clinical trial items, were included in our sample. The 176 respondents excluded because of missing trial invitation status more frequently had less education (eTable 2 in the Supplement). Of the 3689 included respondents, half were female (2107 [50%]), the median (IQR) age was 48 (33-61) years, and most were non-Hispanic White (2063 [59%]; non-Hispanic Black, 452 [10%]; Hispanic, 521 [14%]), had more than a high school degree (2656 [68%]), were employed (1809 [58%]), and had at least 1 medical condition (2535 [61%]) (Table 1).

**Table 1.** Respondent Demographic, Clinical, and Health Behavior–Related Characteristics by Invitation Status

Characteristic	No. (Weighted %)			*V*
	Total (n = 3689)	Invited (n = 439)	Not invited (n = 3250)	
Age				
Weighted median (IQR)	48 (33-61)	54 (41-65)	48 (33-61)	*d* = 0.22
18-34	458 (25.0)	33 (15.9)	425 (26.0)	.08
35-49	680 (25.3)	64 (25.7)	616 (25.2)	
50-64	1105 (27.4)	137 (27.7)	968 (27.4)	
65-74	841 (11.6)	131 (17.1)	710 (11.1)	
≥75	505 (8.2)	62 (9.4)	443 (8.0)	
Missing	100 (2.5)	12 (4.3)	88 (2.3)	
Sex				
Male	1505 (47.7)	154 (44.5)	1351 (48.1)	0.04
Female	2107 (50.4)	274 (51.0)	1833 (50.3)	
Missing	77 (1.9)	11 (4.5)	66 (1.6)	
Race and ethnicity				
Non-Hispanic White	2063 (59.4)	232 (57.2)	1831 (59.6)	0.1
Non-Hispanic Black	452 (10.1)	90 (18.5)	362 (9.3)	
Hispanic	521 (14.2)	49 (11.1)	472 (14.5)	
Other race or multiracial^a^	319 (9.3)	28 (5.8)	291 (9.7)	
Missing	334 (6.9)	40 (7.3)	294 (6.9)	
Education				
<High school	255 (7.5)	23 (4.3)	232 (7.8)	0.07
High school degree	663 (21.7)	53 (14.9)	610 (22.4)	
Some college	1040 (38.6)	135 (37.8)	905 (38.7)	
≥College graduate	1616 (29.7)	214 (39.8)	1402 (28.7)	
Missing	115 (2.5)	14 (3.1)	101 (2.4)	
Feelings about present income				
Living comfortably	1383 (34.4)	150 (30.5)	1233 (34.8)	0.05
Getting by	1388 (39.7)	164 (37.6)	1224 (39.9)	
Finding it difficult	499 (15.0)	67 (15.0)	432 (15.0)	
Finding it very difficult	214 (6.1)	36 (10.6)	178 (5.7)	
Missing	205 (4.8)	22 (6.2)	183 (4.6)	
Marital status				
Married or living as married	1912 (53.6)	201 (48.0)	1711 (54.1)	0.05
Divorced, widowed, or separated	1036 (13.7)	139 (12.8)	897 (13.8)	
Single, never married	620 (29.9)	88 (36.2)	532 (29.3)	
Missing	121 (2.8)	11 (2.9)	110 (2.8)	
Residence				
Rural	299 (8.5)	16 (3.1)	283 (9.0)	0.06
Urban	3390 (91.5)	423 (97.9)	2967 (91.0)	
Region				
Northeast	557 (17.3)	61 (17.2)	496 (17.3)	0.03
Midwest	615 (21.0)	62 (16.9)	553 (21.4)	
South	1643 (37.9)	200 (38.3)	1443 (37.9)	
West	874 (23.8)	116 (27.7)	758 (23.4)	
Employment status				
Employed	1809 (57.9)	178 (49.0)	1631 (58.9)	0.07
Retired	1154 (18.4)	161 (23.2)	993 (17.9)	
Unemployed or receiving disability	321 (9.7)	45 (12.4)	276 (9.5)	
Other	289 (11.4)	36 (10.6)	253 (11.5)	
Missing	116 (2.5)	19 (4.8)	97 (2.3)	
Health insurance status				
Private or employer sponsored	1488 (47.7)	145 (38.5)	1343 (48.6)	0.11
Medicare	1154 (19.4)	178 (24.4)	976 (18.9)	
Medicaid	333 (11.3)	36 (14.7)	297 (10.9)	
Dual eligible	217 (3.8)	41 (9.2)	176 (3.2)	
Other	255 (7.6)	36 (8.3)	228 (7.5)	
Uninsured	194 (9.0)	9 (4.1)	185 (9.5)	
Missing	48 (1.3)	3 (0.8)	45 (1.3)	
Self-reported medical conditions^b^				
Diabetes	789 (17.9)	122 (23.1)	667 (17.4)	0.06
High blood pressure	1627 (35.7)	223 (41.6)	1404 (35.1)	0.05
Heart condition	387 (8.0)	56 (10.3)	331 (7.7)	0.03
Lung disease	526 (12.5)	103 (23.4)	423 (11.4)	0.1
Depression	863 (23.9)	151 (41.7)	712 (22.1)	0.1
Cancer	596 (9.0)	110 (16.2)	486 (8.2)	0.09
No. of medical conditions				
0	1122 (38.4)	76 (17.6)	1046 (40.6)	0.14
1	1145 (30.6)	127 (35.0)	1018 (30.2)	
2	805 (19.1)	113 (26.4)	692 (18.4)	
≥3	585 (11.3)	120 (20.5)	465 (10.3)	
Missing	32 (0.5)	3 (0.6)	29 (0.5)	
Self-rated health				
Excellent or very good	1746 (50.1)	186 (44.6)	1560 (50.6)	0.07
Good	1336 (35.8)	152 (32.2)	1184 (36.2)	
Fair or poor	593 (13.9)	97 (22.2)	496 (13.1)	
Missing	14 (0.2)	4 (0.9)	10 (0.2)	
Current smoker				
Yes	419 (13.6)	60 (14.8)	359 (13.5)	0.03
No	3218 (85.1)	374 (83.5)	2844 (85.3)	
Missing	52 (1.3)	5 (1.7)	47 (1.3)	
Hazardous drinker				
Yes	471 (14.4)	46 (10.4)	425 (14.8)	0.03
No	1227 (32.6)	149 (31.0)	1078 (32.8)	
Missing	1991 (53.0)	244 (58.7)	1747 (52.4)	
Weekly exercise				
None	984 (26.0)	113 (25.1)	871 (26.1)	0.01
Any	2656 (72.8)	319 (74.2)	2337 (72.7)	
Missing	49 (1.2)	7 (0.7)	42 (1.2)	
Saw health care professional in last year				
Yes	3169 (82.2)	415 (93.3)	2754 (81.1)	0.09
No	490 (17.2)	20 (5.1)	470 (18.4)	
Missing	30 (0.6)	4 (1.6)	26 (0.5)	

^a^ Other race included American Indian or Alaska Native, Asian Indian, Chinese, Filipino, Japanese, Korean, Vietnamese, other Asian, Native Hawaiian, Guamanian or Chamorro, Samoan, and other Pacific Islander.

^b^ Will not sum to 100% since respondents could report multiple conditions.

### Characteristics Associated With Clinical Trial Invitation and Participation

Overall, 439 respondents (9%) reported being invited to participate in a clinical trial (Table 1). In exploratory models, respondent factors associated with increased odds of reported clinical trial invitation included being non-Hispanic Black compared with non-Hispanic White (aOR, 1.85; 95% CI, 1.13-3.02), having some college education or a college degree or higher compared with less than high school education (some college: aOR, 2.48; 95% CI, 1.07-5.77; ≥college degree: aOR, 4.84; 95% CI, 1.89-12.39), being single compared with married or living as married (aOR, 1.68; 95% CI, 1.04-2.73), and having at least 1 more medical condition compared with none (1 condition: aOR, 2.25; 95% CI, 1.32-3.82; 2 conditions: aOR, 2.67; 95% CI, 1.56-4.57; ≥3: aOR, 3.76; 95% CI, 2.01-7.03) (Table 2). Conversely, respondents residing in a rural area had 77% decreased odds of invitation to a clinical trial than those residing in an urban area (aOR 0.33; 95% CI 0.17-0.65).

**Table 2.** Multivariable Exploratory Analysis: Odds of Invitation to and Participation in a Clinical Trial by Respondent Demographic, Clinical, and Health Behavior–Related Characteristics

Characteristic	Adjusted odds ratio (95% CI)	
	Odds of invitation (n = 3689)	Odds of participation (n = 429)^a^
Age		
18-34	1 [Reference]	1 [Reference]
35-49	1.61 (0.82-2.92)	1.10 (0.31-3.90)
50-64	1.57 (0.84-2.92)	1.49 (0.53-4.15)
65-74	1.96 (0.98-3.92)	1.16 (0.27-5.10)
≥75	1.71 (0.81-3.62)	2.20 (0.35-13.73)
Sex		
Female	1 [Reference]	1 [Reference]
Male	0.98 (0.63-1.51)	1.14 (0.47-2.75)
Race/ethnicity		
Non-Hispanic White	1 [Reference]	1 [Reference]
Non-Hispanic Black	1.85 (1.13-3.02)	0.28 (0.09-0.87)
Hispanic	1.01 (0.44-2.32)	1.73 (0.57-5.25)
Other race or multiracial^b^	0.51 (0.26-1.00)	0.78 (0.08-7.99)
Education		
<High school	1 [Reference]	1 [Reference]
High school degree	1.52 (0.62-3.74)	2.91 (0.23-37.68)
Some college	2.48 (1.07-5.77)	2.23 (0.13-38.32)
≥College graduate	4.84 (1.89-12.39)	4.81 (0.24-96.38)
Feelings about present income		
Living comfortably	1 [Reference]	1 [Reference]
Getting by	1.16 (0.74-1.82)	1.53 (0.62-3.81)
Finding it difficult	1.13 (0.58-2.21)	1.43 (0.40-5.11)
Finding it very difficult	1.56 (0.57-4.31)	0.69 (0.17-2.85)
Marital status		
Married or living as married	1 [Reference]	NA
Divorced, widowed, separated	0.78 (0.52-1.19)	NA
Single, never married	1.68 (1.04-2.73)	NA
Residence		
Urban	1 [Reference]	1 [Reference]
Rural	0.33 (0.17-0.65)	3.10 (0.64-15.08)
Region		
Northeast	1 [Reference]	NA
Midwest	0.80 (0.41-1.56)	NA
South	1.04 (0.64-1.70)	NA
West	1.55 (0.85-2.85)	NA
Employment status		
Employed	1 [Reference]	NA
Retired	1.09 (0.67-1.77)	NA
Unemployed or receiving disability	0.78 (0.31-1.98)	NA
Other	1.21 (0.63-2.32)	NA
Health insurance status		
Private or employer sponsored	1 [Reference]	1 [Reference]
Medicare	1.17 (0.57-2.41)	1.02 (0.26-4.00)
Medicaid	1.59 (0.79-2.23)	0.33 (0.05-2.43)
Dual eligible	2.65 (0.96-7.32)	0.67 (0.15-3.11)
Other	1.45 (0.53-3.96)	2.37 (0.41-13.58)
Uninsured	1.04 (0.21-5.09)	4.94 (0.07-343.42)
No. of medical conditions		
0	1 [Reference]	NA
1	2.25 (1.32-3.82)	NA
2	2.67 (1.56-4.57)	NA
≥3	3.76 (2.01-7.03)	NA
Self-rated health		
Excellent or very good	1 [Reference]	1 [Reference]
Good	0.98 (0.62-1.55)	0.87 (0.33-2.27)
Fair or poor	1.43 (0.80-2.55)	1.29 (0.52-3.20)
Current smoker		
Yes	1.30 (0.73-2.32)	NA
No	1 [Reference]	NA
Hazardous drinker		
No	1 [Reference]	NA
Yes	0.91 (0.53-1.56)	NA
Weekly exercise		
Any	1 [Reference]	NA
None	0.87 (0.58-1.32)	NA
Saw health care professional in last year		
No	1 [Reference]	NA
Yes	2.74 (1.33-5.65)	NA

Abbreviation: NA, not applicable.

^a^ A total of 10 respondents reported invitation but had missing participation status.

^b^ Other race included American Indian or Alaska Native, Asian Indian, Chinese, Filipino, Japanese, Korean, Vietnamese, other Asian, Native Hawaiian, Guamanian or Chamorro, Samoan, and other Pacific Islander.

Of those invited to a clinical trial, 199 respondents (47%) reported participating (Table 3). In models estimating odds of reported clinical trial participation, respondents who were non-Hispanic Black compared with non-Hispanic White had 72% decreased odds of participation after invitation (aOR, 0.28; 95% CI, 0.09-0.87) (Table 2). In sensitivity analyses excluding 1345 respondents who reported knowing nothing about clinical trials, education and marital status were no longer associated with trial invitation, while dual-eligible beneficiaries had higher odds of invitation compared with those with private insurance (eTable 3 in the Supplement). Trial participation model results were similar.

**Table 3.** Invited Respondent Demographic, Clinical, and Health Behavior–Related Characteristics by Participation Status

Characteristic	No. (Weighted %)			*V*
	Invited (n = 439)^a^	Participated (n = 199)	Did not participate (n = 230)	
Age				
Weighted median (IQR)	54 (41-65)	53 (39-66)	54 (41-65)	*d* = 0.08
18-34	33 (15.9)	12 (17.8)	20 (14.0)	.08
35-49	64 (25.7)	30 (23.0)	34 (29.2)	
50-64	137 (27.7)	63 (27.3)	72 (27.6)	
65-74	131 (17.1)	62 (15.8)	65 (18.1)	
≥75	62 (9.4)	29 (11.3)	32 (7.8)	
Missing	12 (4.3)	3 (4.8)	7 (3.2)	
Sex				
Male	154 (44.5)	67 (44.0)	84 (45.0)	0.03
Female	274 (51.0)	128 (50.1)	141 (52.5)	
Missing	11 (4.5)	4 (5.9)	5 (2.4)	
Race and ethnicity				
Non-Hispanic White	232 (57.2)	123 (64.3)	107 (52.6)	0.17
Non-Hispanic Black	90 (18.5)	30 (10.0)	57 (24.4)	
Hispanic	49 (11.1)	22 (14.8)	26 (8.2)	
Other race or multiracial^b^	28 (5.8)	8 (5.3)	18 (6.1)	
Missing	40 (7.3)	16 (5.5)	22 (8.6)	
Education				
<High school	23 (4.3)	4 (1.3)	14 (4.1)	0.15
High school degree	53 (14.9)	23 (13.5)	29 (16.6)	
Some college	135 (37.8)	54 (13.5)	81 (16.6)	
≥College graduate	214 (39.8)	111 (33.7)	100 (43.2)	
Missing	14 (3.1)	7 (3.5)	6 (2.7)	
Feelings about present income				
Living comfortably	150 (30.5)	73 (31.0)	73 (30.0)	0.06
Getting by	164 (37.6)	74 (41.3)	89 (35.9)	
Finding it difficult	67 (15.0)	28 (15.8)	35 (12.8)	
Finding it very difficult	36 (10.6)	16 (5.6)	20 (15.5)	
Missing	22 (6.2)	8 (6.4)	13 (5.8)	
Marital status				
Married or living as married	201 (48.0)	100 (53.4)	95 (43.6)	0.09
Divorced, widowed, or separated	139 (12.8)	59 (12.0)	80 (14.2)	
Single, never married	88 (36.2)	35 (31.4)	50 (39.7)	
Missing	11 (2.9)	5 (3.2)	5 (2.5)	
Residence				
Rural	16 (3.1)	12 (4.9)	4 (1.7)	0.11
Urban	423 (97.9)	187 (95.1)	226 (98.3)	
Region				
Northeast	61 (17.2)	27 (13.0)	31 (20.0)	0.05
Midwest	62 (16.9)	29 (18.2)	30 (15.5)	
South	200 (38.3)	87 (38.6)	111 (38.1)	
West	116 (27.7)	56 (30.3)	58 (26.3)	
Employment status				
Employed	178 (49.0)	75 (52.4)	100 (47.7)	0.11
Retired	161 (23.2)	81 (25.9)	77 (21.1)	
Unemployed or receiving disability	45 (12.4)	19 (7.1)	25 (16.9)	
Other	36 (10.6)	19 (9.6)	16 (10.5)	
Missing	19 (4.8)	5 (5.0)	12 (3.8)	
Health insurance status				
Private or employer sponsored	145 (38.5)	68 (41.8)	75 (35.3)	0.16
Medicare	178 (24.4)	88 (25.5)	86 (23.8)	
Medicaid	36 (14.7)	7 (6.8)	27 (21.8)	
Dual eligible	41 (9.2)	19 (6.5)	22 (11.9)	
Other	36 (8.3)	11 (12.7)	14 (3.6)	
Uninsured	9 (4.1)	5 (6.5)	4 (2.2)	
Missing	3 (0.8)	1 (0.3)	2 (1.3)	
Self-reported medical conditions^c^				
Diabetes	122 (23.1)	53 (23.3)	66 (23.3)	0.05
High blood pressure	223 (41.6)	95 (38.8)	124 (45.0)	0.08
Heart condition	56 (10.3)	28 (10.2)	26 (10.4)	0.06
Lung disease	103 (23.4)	44 (22.0)	58 (25.5)	0.04
Depression	151 (41.7)	70 (37.8)	79 (45.7)	0.01
Cancer	110 (16.2)	46 (13.0)	63 (19.5)	0.06
No. of medical conditions				
0	76 (17.6)	43 (22.9)	29 (11.2)	0.13
1	127 (35.0)	50 (30.5)	74 (39.1)	
2	113 (26.4)	51 (28.6)	60 (25.2)	
≥3	120 (20.5)	54 (17.5)	65 (23.8)	
Missing	3 (0.6)	1 (0.4)	2 (0.7)	
Self-rated health				
Excellent or very good	186 (44.6)	94 (47.5)	87 (42.6)	0.11
Good	152 (32.2)	66 (30.1)	83 (33.2)	
Fair or poor	97 (22.2)	38 (21.4)	57 (23.4)	
Missing	4 (0.9)	1 (1.1)	3 (0.8)	
Current smoker				
Yes	60 (14.8)	29 (13.2)	29 (15.0)	0.1
No	374 (83.5)	170 (86.8)	196 (81.6)	
Missing	5 (1.7)	0 (0.0)	5 (3.3)	
Hazardous drinker				
Yes	46 (10.4)	22 (9.0)	22 (10.2)	0.03
No	149 (31.0)	69 (31.9)	79 (31.0)	
Missing	244 (58.7)	108 (59.1)	129 (58.7)	
Weekly exercise				
None	113 (25.1)	47 (22.0)	65 (28.7)	0.13
Any	319 (74.2)	152 (78.0)	158 (69.8)	
Missing	7 (0.7)	0 (0.0)	7 (1.4)	
Saw health care professional in last year				
Yes	415 (93.3)	191 (94.9)	216 (93.0)	0.08
No	20 (5.1)	8 (5.1)	11 (5.3)	
Missing	4 (1.6)	0 (0.0)	3 (1.7)	

^a^ A total of 10 respondents reported invitation but were missing participation status.

^b^ Other race included American Indian or Alaska Native, Asian Indian, Chinese, Filipino, Japanese, Korean, Vietnamese, other Asian, Native Hawaiian, Guamanian or Chamorro, Samoan, and other Pacific Islander.

^c^ Will not sum to 100% since respondents could report multiple conditions.

### Information Sources, Trust, and Motives for Clinical Trial Participation Among All Respondents

**Figure 1.**
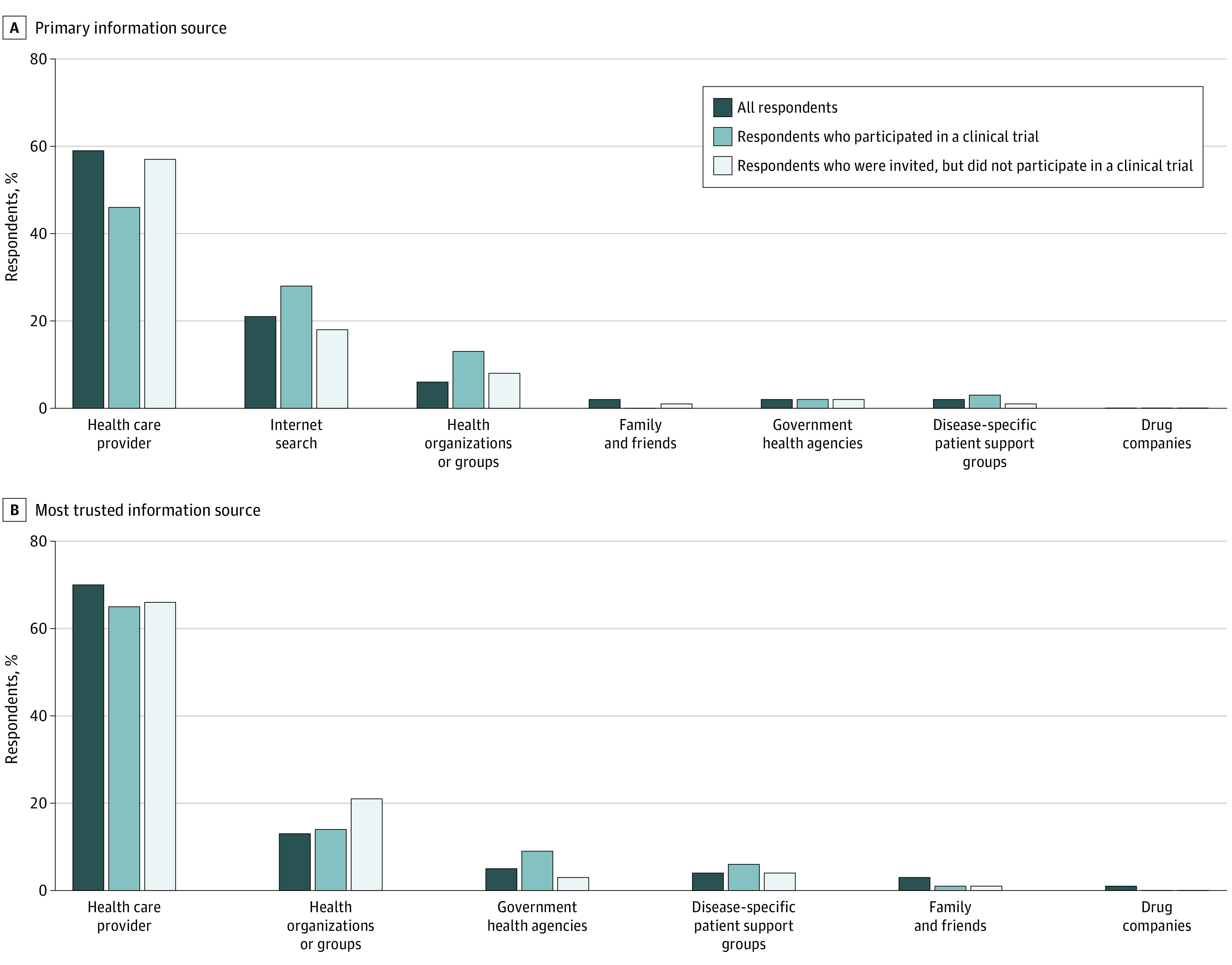
Respondent-Reported Primary and Most Trusted Information Source on Clinical Trials All respondents include 3689 individuals; respondents who participated in a clinical trial, 199 individuals; respondents who were invited, but did not participate in a clinical trial, 230 individuals.

When respondents were asked to imagine a need for clinical trial information, “health care providers” were most frequently reported as their first source of information (2297 [59%]), followed by the internet (644 [21%]) (Figure 1A). “Health care providers” were also reported most frequently as respondents most trusted information source (2597 [70%]), followed by health organizations or groups (456 [13%]) (Figure 1B). Among those invited to a clinical trial, primary information sources differed between those who did and did not participate. Respondents who reported participating in clinical trials, compared with those who did not report participation, less frequently reported physicians (94 of 199 [46%] vs 145 of 230 [57%]) and more frequently reported the internet as a first source of information (44 [28%] vs 34 [18%]). Among those invited to a clinical trial, trust in information sources did not differ substantially between those who did and did not participate.

**Figure 2.**
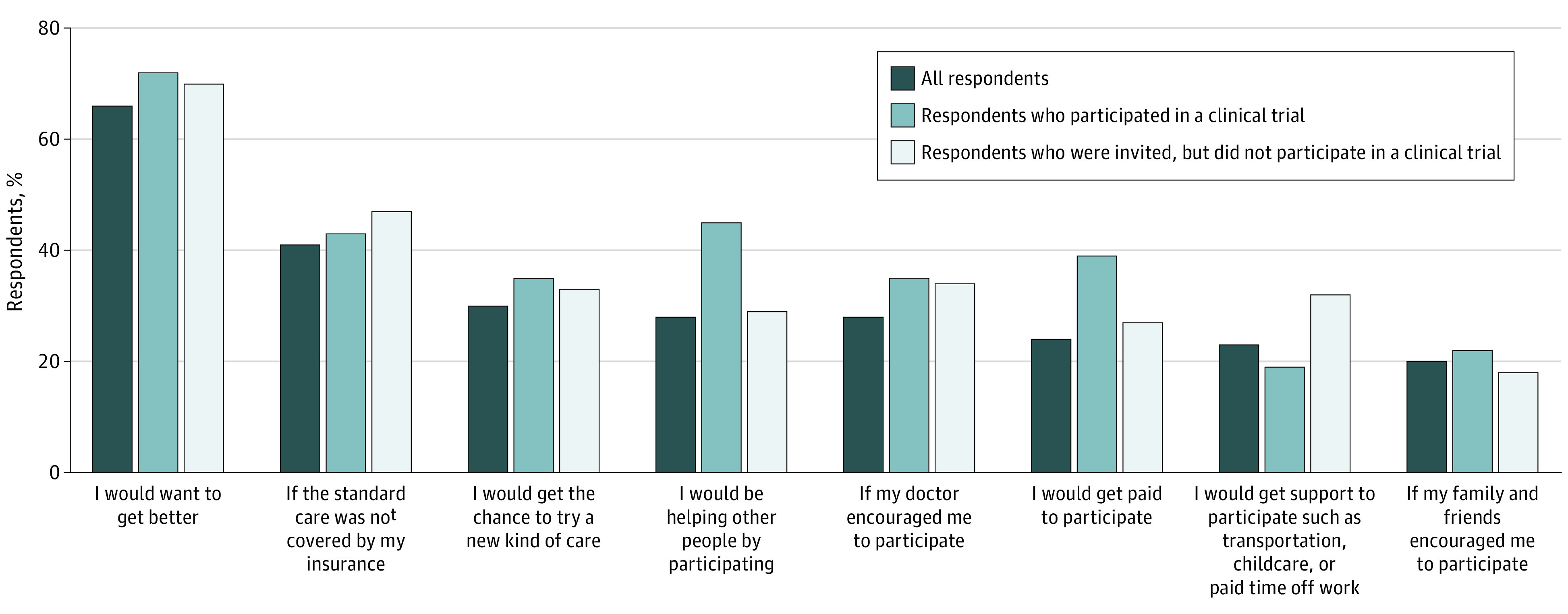
Respondent Ranking of Factors Motivating Participation in Clinical Trials All respondents include 3689 individuals; respondents who participated in a clinical trial, 199 individuals; respondents who were invited, but did not participate in a clinical trial, 230 individuals.

When all respondents were asked to imagine being invited to a clinical trial for any health issue, the most influential factors in the decision to participate in a trial included “wanting to get better” (2294 [66%]), the standard of care not being covered by insurance (1448 [41%]), and the chance to try a new kind of care (1085 [30%] (Figure 2). Among those invited to a clinical trial, influential factors in the decision to participate differed between those who did and did not participate. Respondents who reported participating in trials, compared with those who did not report participation, were more motivated by helping other people (98 of 199 [45%] vs 70 of 230 [29%]) and getting paid (63 [39%] vs 49 [27%]).

## Discussion

Results of this nationally representative survey suggest that approximately 1 in 10 Americans report being invited to participate in a clinical trial, with a little less than half of those invited reporting participation. In multivariable models, respondents had higher odds of clinical trial invitation if they were non-Hispanic Black, had a college education, were single, were urban-dwelling, or had at least 1 medical condition. However, non-Hispanic Black respondents had lower odds of self-reported trial participation than non-Hispanic White respondents. These results reinforce the need for future research promoting effective clinical trial communication via health care professionals and the internet, as these were identified as important sources of clinical trial information. Our results also highlight a need for further research on ameliorative, financial, and altruistic factors as potential engagement strategies for trial participation.

Non-Hispanic Black respondents in our study had higher odds of reported invitation to clinical trials than non-Hispanic White respondents. These results contrast with prior literature proposing trial design- and researcher-level explanations for racial and ethnic disparities in clinical trial representation, such as restrictive eligibility criteria and researcher bias, that may hinder equitable invitation.^31,32,33^ Our results may reflect researcher-level uptake of initiatives to increase the diversity of clinical trial participants. Since the 1993 National Institutes of Health (NIH) Revitalization Act^34^ mandating inclusion of racial and ethnic minority groups in NIH-funded research, increased attention has been placed on greater clinical trial inclusivity.^35,36^ More recently, the US Food and Drug Administration (FDA) issued recommendations for greater inclusion of certain segments of the population, including those identifying as Black, Indigenous, or another minority racial or ethnic group as well as analysis of study results by race and ethnicity.^37^ Continued commitment to equity in trial invitation may help ensure equitable representation in clinical trials.

Despite greater odds of reported invitation to clinical trials in this study, non-Hispanic Black respondents had lower odds of reported participation in clinical trials than non-Hispanic White respondents. This contrasts with previous work showing Black and White individuals enroll in clinical trial at similar rates if invited.^30,31,38,39^ Although our study controlled for comorbidity status, health care engagement, and financial burden, other items influencing barriers to clinical trial participation, such as ineligibility or lack of access, may result in lower participation rates. Furthermore, historical research mistreatment toward the Black community, current disparities in health care access and quality, and negative encounters with health care professionals may also affect trial participation.^40,41^ Multilevel institutional efforts addressing eligibility, access, and systemic racism are needed to decrease racial and ethnic health inequities in clinical trial participation, such as diversifying the health care workforce, increasing community engagement, enhancing transparency of research practices, and training health care professionals in structural competency, defined as identifying and clinically addressing structural drivers of health inequity.^42,43,44,45^ Building an environment of trust and facilitating effective communication between patients and the health care system could also be helpful. Participation in therapeutic cancer clinical trials nearly doubled among Black patients receiving trial education by nonclinical patient navigators compared with patients not receiving navigation services.^46^ Other multilevel efforts, such as information dissemination via community health advisors,^47^ warrant further research.

In our study, respondents residing in rural compared with urban areas had decreased odds of invitation to clinical trials. These results may stem from the historical lack of trial availability for individuals receiving medical care in rural settings. Recent efforts have focused on remediating this discrepancy. Current NIH-funded initiatives expand trial availability in community-based settings by dismantling patient-level barriers to trial participation related to residing far from care settings. The National Cancer Institute Community Oncology Research Program,^29,48^ the National Institute on Drug Abuse Clinical Trials Network,^49^ and the NIH Collaboratory Distributed Research Network^50^ bring more research into community settings, offering increased opportunities for participation in clinical trials closer to their residence. To ensure equitable access to trials, increased awareness of trial availability is needed, especially among underserved and diverse patient populations.

Health care professionals may serve as the greatest opportunity to increase clinical trial enrollment, given that they were both the first and most trusted clinical trial information source reported by 59% and 70% of all respondents, respectively. This suggests patient-clinician communication is key to raising patient awareness of and participation in clinical trials. Clinicians must first be aware of actively recruiting trials as well as their eligibility criteria to communicate potential enrollment with their patients.^51,52,53^ Furthermore, communicating complex trial protocols to patients with differing health literacy is challenging because of time-related constraints during clinical encounters.^33^ The internet was reported as a trustworthy source of information by 28% of respondents, suggesting future research in web-based education and counseling to increase efficiency of in-clinic trial communications.^54^ Culturally appropriate, plain-language tools could also facilitate patient-clinician communication, especially for patients without internet access or with low health literacy.^55,56^ Historically underrepresented potential research participants found simple paper flashcards to be effective communication tools given that they quickly communicate research information.^57^ Other modalities, like video, do not increase trial knowledge, but help facilitate extended discussions between patients and clinicians about clinical trial participation.^58,59^

Financial incentives were commonly reported by study respondents as influential in their clinical trial participation decision, including having treatment payment covered and getting paid for trial participation. Although payment for treatment and routine care received while participating in trials may act as a facilitator to clinical trial participation, indirect costs related to trial participation, such as transportation to the clinic or time off work to participate, may act as barriers. Barriers related to indirect trial expenses may explain why respondents enrolled in Medicaid or dually enrolled were more often invited to trials but less often participated. Our results are consistent with previous studies regarding patient financial issues surrounding trial participation.^27,60,61^ To potentially remove financial barriers to participation, recent recommendations from advocacy, research, and governmental organizations, including the FDA, have encouraged patient reimbursement for direct and indirect trial-related costs.^62,63,64,65,66^ In December 2020, Congress passed the Clinical Treatment Act, which requires Medicaid to cover routine clinical care costs associated with clinical trial participation,^67^ removing some financial barriers and furthering representation of disparate patient groups. However, quantification of and reimbursement for indirect trial costs in a trial environment remains unclear. Furthermore, trialists should ensure financial incentives are ethical and do not result in coercion or undue influence for study participation.^68^ Efforts to reduce financial hardship for individuals willing to participate in clinical trials should be considered an ethical priority for trialists to increase equitable access for all.

### Limitations

The results from our study should be considered within the context of several limitations. The data for this study were subject to selection biases including mailed survey coverage error, nonresponse, and voluntary response potentially associated with likelihood to participate in a clinical trial. However, HINTS data weights were specifically calibrated to compensate for these biases.^69^ Data were self-reported by HINTS respondents and therefore subject to recall bias. Although items were developed using 2 rounds of cognitive testing to maximize understandability, variation in respondent interpretation of these questions is possible. In particular, rates of invitation to clinical trials may be overreported in the current data, as respondents may have difficulty differentiating invitation to a clinical trial from prescreening or eligibility assessment for a trial, a more general discussion of a trial, or even invitation to other health research (eg, survey or qualitative studies). Future efforts to decrease measurement error are needed for the development of interventions targeting equitable clinical trial invitation and enrollment. The complexity surrounding the multilevel factors influencing clinical trial invitation and participation was not captured. Our results may be influenced by the low general knowledge of clinical trials in our sample, especially since this survey was fielded outside of a usual care setting. However, sensitivity analyses excluding respondents reporting no knowledge of clinical trials revealed similar results in our multivariable models. Respondents reported financial access to standard of care medications as a primary motivator to trial participation. This may be a uniquely US phenomenon given the high cost of health care, even for individuals with insurance. Because our survey was fielded during spring 2020, results may also be influenced by increased awareness of trials by the US public due to the COVID-19 pandemic. However, rates of invitation and participation were similar for surveys returned both before and after March 11, 2020, the date the pandemic was declared. Additionally, our results are not disease specific but rather provide a generalized snapshot of clinical trial engagement nationwide.

## Conclusions

In this nationally representative sample, approximately 1 in 10 respondents reported being invited to participate in a clinical trial, and nearly half of those respondents reported participating in a trial after invitation. Self-reported clinical trial invitation was associated with respondent race and ethnicity, residence, education, marital status, and comorbidity status, while self-reported trial participation was associated with race and ethnicity alone. Respondents reported “health care providers” and the internet as important sources of clinical trial information, and health improvement, financial incentives, and altruism as key motivations for clinical trial participation. Strategies for increasing trial participation rates across diverse patient populations, such as increasing trial availability across diverse geographic care settings, facilitating patient-clinician trial information communication, and providing financial reimbursement for trial participation, warrant further research to ensure equitable translation of clinical benefits from research to practice.

## Notes

Supplement. **eTable 1.** Respondent Ranking of Factors Motivating Participation in Clinical Trials **eTable 2.** Demographic, Clinical, and Health Behavior–Related Characteristics for Respondents Excluded Due to Missing Clinical Trial Invitation Status and Included In Complete Case Analysis **eTable 3. **Multivariable Exploratory Analysis Excluding Respondents Who Reported Knowing Nothing About Clinical Trials: Odds of Invitation to and Participation in a Clinical Trial by Respondent Demographic, Clinical, and Health Behavior–Related Characteristics **eFigure.** Health Information National Trends Survey (HINTS) 5 Cycle 4 Survey Tool: Clinical Trial Questions
