# Supplementary material for: Demographic and Health Behavior Factors Associated With Clinical Trial Invitation and Participation in the United States
Source: JAMA Netw Open. 2021 Sep 29;4(9):e2127792. doi: 10.1001/jamanetworkopen.2021.27792 (PMC8482053; doi:10.1001/jamanetworkopen.2021.27792)
Supplement: Supplement. — eTable 1. Respondent Ranking of Factors Motivating Participation in Clinical Trials eTable 2. Demographic, Clinical, and Health Behavior–Related Characteristics for Respondents Excluded Due to Missing Clinical Trial Invitation Status and Included In Complete Case Analysis eTable 3. Multivariable Exploratory Analysis Excluding Respondents Who Reported Knowing Nothing About Clinical Trials: Odds of Invitation to and Participation in a Clinical Trial by Respondent Demographic, Clinical, and Health Behavior–Related Characteristics eFigure. Health Information National Trends Survey (HINTS) 5 Cycle 4 Survey Tool: Clinical Trial Questions [file jamanetwopen-e2127792-s001.pdf]

Supplemental Online Content

Williams CP, Senft Everson N, Shelburne N, Norton WE. Demographic and health behavior factors associated with clinical trial invitation and participation in the United States. *JAMA Netw Open.* 2021;4(9):e2127792. doi:10.1001/jamanetworkopen.2021.27792

eTable 1. Respondent Ranking of Factors Motivating Participation in Clinical Trials

eTable 2. Demographic, Clinical, and Health Behavior–Related Characteristics for Respondents Excluded Due to Missing Clinical Trial Invitation Status and Included In Complete Case Analysis

eTable 3. Multivariable Exploratory Analysis Excluding Respondents Who Reported Knowing Nothing About Clinical Trials: Odds of Invitation to and Participation in a Clinical Trial by Respondent Demographic, Clinical, and Health Behavior–Related Characteristics

eFigure. Health Information National Trends Survey (HINTS) 5 Cycle 4 Survey Tool: Clinical Trial Questions

This supplemental material has been provided by the authors to give readers additional information about their work.

© 2021 Williams CP et al. *JAMA Network Open.*

eTable 1. Respondent Ranking of Factors Motivating Participation in Clinical Trials

	All respondents (N=3,689)				Respondents who participated in a clinical trial (n=199)				Respondents who were invited, but did not participate in a clinical trial (n=230)			
	A lot	Somewhat	A little	Not at all	A lot	Somewhat	A little	Not at all	A lot	Somewhat	A little	Not at all
I would want to get better.	66%	20%	7%	5%	72%	22%	3%	2%	70%	11%	6%	5%
If the standard care was not covered by my insurance.	41%	25%	14%	17%	43%	39%	9%	8%	47%	24%	9%	14%
I would get the chance to try a new kind of care.	30%	35%	20%	12%	35%	38%	15%	12%	33%	33%	19%	9%
I would be helping other people by participating.	28%	38%	21%	10%	45%	42%	5%	7%	29%	36%	21%	6%
If my doctor encouraged me to participate.	28%	37%	21%	11%	35%	44%	16%	5%	34%	33%	22%	3%
I would get paid to participate.	24%	28%	22%	23%	39%	28%	15%	16%	27%	27%	24%	16%
I would get support to participate such as transportation, childcare, or paid time off work.	23%	28%	21%	25%	19%	30%	22%	28%	32%	15%	15%	29%
If my family and friends encouraged me to participate.	20%	37%	24%	15%	22%	44%	26%	7%	18%	34%	28%	11%

© 2021 Williams CP et al. *JAMA Network Open.*

eTable 2. Demographic, Clinical, and Health Behavior–Related Characteristics for Respondents Excluded Due to Missing Clinical Trial Invitation Status and Included In Complete Case Analysis (N=3,865).

	Excluded due to missing invitation status n=176	Included in complete case analysis n=3,689
	n (Weighted %)	n (Weighted %)
Age (weighted median, IQR)	45 (31-66)	48 (33-61)
18-34	26 (35.5)	458 (25.0)
35-49	23 (13.9)	680 (25.3)
50-64	37 (16.3)	1105 (27.4)
65-74	28 (11.0)	841 (11.6)
≥75	35 (13.0)	505 (8.2)
Missing	27 (10.4)	100 (2.5)
Sex		
Male	56 (44.0)	1505 (47.7)
Female	97 (46.0)	2107 (50.4)
Missing	23 (9.9)	77 (1.9)
Race/ethnicity		
Non-Hispanic White	70 (42.2)	2063 (59.4)
Non-Hispanic Black	29 (15.1)	452 (10.1)
Hispanic	25 (12.1)	521 (14.2)
Other race/multiracial	11 (13.5)	319 (9.3)
Missing	41 (17.0)	334 (6.9)
Education		
Less than high school	18 (14.9)	255 (7.5)
High school degree	42 (26.8)	663 (21.7)
Some college	41 (26.1)	1040 (38.6)
College graduate or higher	47 (22.5)	1616 (29.7)
Missing	28 (9.7)	115 (2.5)
Feelings about present income		
Comfortable	49 (34.4)	1383 (34.4)
Getting by	60 (31.3)	1388 (39.7)
Finding it difficult	29 (16.2)	499 (15.0)
Finding it very difficult	10 (8.2)	214 (6.1)
Missing	28 (10.0)	205 (4.8)
Marital status		
Married/living as married	66 (43.3)	1912 (53.6)
Divorced/widowed/separated	62 (19.9)	1036 (13.7)
Single, never married	25 (28.6)	620 (29.9)
Missing	23 (8.1)	121 (2.8)
Residence		
Rural	12 (5.0)	299 (8.5)
Urban	164 (95.0)	3390 (91.5)
Region		
Northeast	24 (23.6)	557 (17.3)
Midwest	30 (17.5)	615 (21.0)
South	85 (37.3)	1643 (37.9)
West	37 (21.6)	874 (23.8)
Employment status		
Employed	64 (41.8)	1809 (57.9)
Retired	52 (17.8)	1154 (18.4)
Unemployed/disabled	21 (12.5)	321 (9.7)

© 2021 Williams CP et al. *JAMA Network Open.*

	Excluded due to missing invitation status<br>n=176	Included in complete case analysis<br>n=3,689
	n<br>(Weighted %)	n<br>(Weighted %)
Other	13 (17.0)	289 (11.4)
Missing	26 (10.9)	116 (2.5)
Health insurance status		
Private/employer sponsored	59 (45.9)	1488 (47.7)
Medicare	43 (14.1)	1154 (19.4)
Medicaid	19 (14.9)	333 (11.3)
Dual eligible	23 (8.0)	217 (3.8)
Other	13 (8.8)	255 (7.6)
Uninsured	9 (5.7)	194 (9.0)
Missing	10 (2.5)	48 (1.3)
Self-reported medical conditions		
Diabetes	36 (12.3)	789 (17.9)
High blood pressure	71 (36.0)	1627 (35.7)
Heart condition	20 (9.6)	387 (6.8)
Lung disease	30 (15.2)	526 (12.5)
Depression	45 (28.3)	863 (23.9)
Cancer	30 (10.7)	596 (9.0)
Number of medical conditions		
0	34 (28.1)	1122 (38.4)
1	51 (31.9)	1145 (30.6)
2	28 (12.6)	805 (19.1)
≥3	32 (15.2)	585 (11.3)
Missing	31 (12.1)	32 (0.5)
Self-rated health		
Excellent/very good	48 (30.4)	1746 (50.0)
Good	62 (40.7)	1336 (35.8)
Fair/poor	34 (16.5)	593 (13.9)
Missing	32 (12.4)	14 (0.2)
Current smoker		
Yes	17 (14.1)	419 (13.6)
No	139 (76.4)	3218 (85.1)
Missing	20 (9.5)	52 (1.3)
Hazardous drinker		
Yes	19 (9.5)	471 (14.4)
No	44 (29.0)	1227 (32.6)
Missing	113 (48.3)	1991 (53.0)
Weekly exercise		
None	64 (42.7)	984 (26.1)
Any	94 (49.2)	2656 (72.8)
Missing	18 (8.1)	49 (1.2)
Saw provider in last year		
Yes	150 (92.3)	3169 (82.2)
No	20 (6.6)	490 (17.2)
Missing	6 (1.1)	30 (0.6)

IQR=interquartile range

© 2021 Williams CP et al. *JAMA Network Open.*

eTable 3. Multivariable Exploratory Analysis Excluding Respondents Who Reported Knowing Nothing About Clinical Trials: Odds of Invitation to and Participation in a Clinical Trial by Respondent Demographic, Clinical, and Health Behavior–Related Characteristics (N=2,283).

	Odds of invitation N=2,283	Odds of participation N=368*
	Adjusted Odds Ratio (95% Confidence Interval)	Adjusted Odds Ratio (95% Confidence Interval)
Age		
18-34	1 [Reference]	1 [Reference]
35-49	1.32 (0.65-2.68)	1.68 (0.29-9.71)
50-64	1.47 (0.77-2.82)	1.75 (0.48-6.36)
65-74	1.54 (0.73-3.26)	0.98 (0.10-9.94)
≥75	1.24 (0.52-2.96)	1.18 (0.11-12.20)
Sex		
Male	0.95 (0.60-1.53)	1.50 (0.42-5.11)
Female	1 [Reference]	1 [Reference]
Race/ethnicity		
Non-Hispanic White	1 [Reference]	1 [Reference]
Non-Hispanic Black	2.35 (1.32-4.18)	0.23 (0.07-0.72)
Hispanic	1.18 (0.40-3.49)	1.47 (0.42-5.11)
Other race/multiracial	0.59 (0.29-1.23)	0.67 (0.08-5.50)
Education		
Less than high school	1 [Reference]	1 [Reference]
High school degree	1.66 (0.21-2.08)	1.82 (0.12-27.45)
Some college	1.26 (0.42-3.80)	2.11 (0.09-47.11)
College graduate or higher	2.26 (0.68-7.53)	5.70 (0.26-123.79)
Feelings about present income		
Living comfortably on present income	1 [Reference]	1 [Reference]
Getting by on present income	1.23 (0.78-1.95)	1.11 (0.40-3.09)
Finding it difficult on present income	1.23 (0.63-2.43)	0.95 (0.15-6.26)
Finding it very difficult on present income	1.65 (0.51-5.34)	0.64 (0.12-3.40)
Marital status		
Married/living as married	1 [Reference]	--
Divorced/widowed/separated	0.92 (0.56-1.52)	--
Single, never married	1.56 (0.92-2.64)	--
Residence		
Rural	0.44 (0.21-0.92)	2.63 (0.55-12.68)
Urban	1 [Reference]	1 [Reference]
Region		
Northeast	1 [Reference]	--
Midwest	1.16 (0.58-2.34)	--
South	1.17 (0.69-1.98)	--
West	2.28 (1.21-4.26)	--
Employment status		
Employed	1 [Reference]	--
Retired	1.09 (0.63-1.90)	--
Unemployed/disabled	0.64 (0.24-1.70)	--
Other	1.82 (0.96-3.43)	--
Health insurance status		
Private/employer sponsored	1 [Reference]	1 [Reference]

© 2021 Williams CP et al. *JAMA Network Open.*

	Odds of invitation N=2,283	Odds of participation N=368*
	Adjusted Odds Ratio (95% Confidence Interval)	Adjusted Odds Ratio (95% Confidence Interval)
Medicare	1.45 (0.57-3.66)	2.38 (0.34-16.90)
Medicaid	1.00 (0.45-2.21)	1.25 (0.14-11.41)
Dual eligible	4.17 (1.32-13.22)	1.16 (0.12-11.08)
Other	1.79 (0.59-5.42)	2.65 (0.51-13.85)
Uninsured	1.09 (0.37-3.27)	2.57 (0.12-54.99)
Number of medical conditions		
0	1 [Reference]	--
1	1.87 (1.06-3.32)	--
2	2.71 (1.51-4.88)	--
≥3	3.68 (2.05-6.58)	--
Self-rated health		
Excellent/very good	1 [Reference]	1 [Reference]
Good	1.00 (0.57-1.75)	1.16 (0.40-3.38)
Fair/poor	1.60 (0.71-3.61)	1.03 (0.25-4.26)
Current smoker		
Yes	1.19 (0.56-2.54)	--
No	1 [Reference]	--
Hazardous drinker		
Yes	0.64 (0.38-1.08)	--
No	1 [Reference]	--
Weekly exercise		
None	0.83 (0.50-1.37)	--
Any	1 [Reference]	--
Saw provider in last year		
Yes	3.08 (1.40-6.84)	--
No	1 [Reference]	--

*n=8 respondents reported invitation but missing participation status.

© 2021 Williams CP et al. *JAMA Network Open.*

G: Clinical Trials

G1. Clinical trials are research studies that involve people. They are designed to compare new kinds of health care with the standard health care people currently get. For example, a new drug or a new way for patients to track their diets.

How would you describe your level of knowledge about clinical trials?
ClinicalTrialKnowledge

- 1 I don't know anything about clinical trials
- 2 I know a little bit about clinical trials
- 3 I know a lot about clinical trials

G2. Imagine that you had a health issue and you were invited to participate in a clinical trial for that issue. How much would each of the following influence your decision to participate in the clinical trial?

	Not at all	A little	Somewhat	A lot
a. I would be helping other people by participating........................ ClinTrial_HelpingPeople	4	3	2	1
b. I would get paid to participate.................................. ClinTrial_GetPaid	4	3	2	1
c. I would get support to participate such as transportation, childcare, or paid time off from work............... ClinTrial_GetSupport	4	3	2	1
d. If my doctor encouraged me to participate.................................. ClinTrial_DocEncouraged	4	3	2	1
e. If my family and friends encouraged me to participate.... ClinTrial_FamEncouraged	4	3	2	1
f. I would want to get better........... ClinTrial_GetBetter	4	3	2	1
g. I would get the chance to try a new kind of care........................ ClinTrial_NewCare	4	3	2	1
h. If the standard care was not covered by my insurance........... ClinTrial_StdNotCovered	4	3	2	1

G3. Imagine you had a need to get information about clinical trials. Which of the following would you go to <u>first</u> to get information about clinical trials?
FirstInfoClinTrials

Mark only <u>one</u>.

- 1 My health care provider
- 2 My family and friends
- 3 Government health agencies
- 4 Health organizations or groups (for example, the American Cancer Society, American Lung Association)
- 5 Disease-specific patient support groups
- 6 Drug companies
- 7 Internet search

FirstInfoClinTrials_IMP

G4. Imagine you had a need to get information about clinical trials. Which of the following <u>would you most trust</u> as a source of information about clinical trials?
TrustInfoClinTrials

Mark only <u>one</u>.

- 1 My health care provider
- 2 My family and friends
- 3 Government health agencies
- 4 Health organizations or groups (for example, the American Cancer Society, American Lung Association)
- 5 Disease-specific patient support groups
- 6 Drug companies

TrustInfoClinTrials_IMP

G5. Have you ever heard of the website clinicaltrials.gov?
HeardClinTrialsWebsite

- 1 Yes
- 2 No

19011

© 2021 Williams CP et al. *JAMA Network Open.*

G6. Have you ever been invited to participate in a clinical trial?
InvitedClinTrial
- 1 Yes
- 2 No → **GO TO H1 below**
- 3 I don't know/I don't remember → **GO TO H1 below**

G7. Did you participate in the clinical trial?
ParticipatedClinTrial
- 1 Yes
- 2 No
- 3 I don't remember

H: Your Overall Health

H1. In general, would you say your health is...
GeneralHealth
- 1 Excellent,
- 2 Very good,
- 3 Good,
- 4 Fair, or
- 5 Poor?

H2. Overall, how confident are you about your ability to take good care of your health?
OwnAbilityTakeCareHealth
- 1 Completely confident
- 2 Very confident
- 3 Somewhat confident
- 4 A little confident
- 5 Not confident at all

H3. Are you deaf or do you have serious difficulty hearing?
Deaf
- 1 Yes
- 2 No

H4. Do you have friends or family members that you talk to about your health?
TalkHealthFriends
- 1 Yes
- 2 No

H5. Has a doctor or other health professional ever told you that you had any of the following medical conditions:

	Yes	No
a. Diabetes or high blood sugar? MedConditions_Diabetes	1	2
b. High blood pressure or hypertension? MedConditions_HighBP	1	2
c. A heart condition such as heart attack, angina, or congestive heart failure? MedConditions_HeartCondition	1	2
d. Chronic lung disease, asthma, emphysema, or chronic bronchitis? MedConditions_LungDisease	1	2
e. Depression or anxiety disorder? MedConditions_Depression	1	2

H6. About how tall are you without shoes?

☐ Feet ***and*** ☐☐ Inches

Height_Feet, Height_Inches

H7. About how much do you weigh, in pounds, without shoes?

☐☐☐ Weight Pounds

H8. <u>Over the past 2 weeks</u>, how often have you been bothered by any of the following problems?

	Nearly every day	More than half the days	Several days	Not at all
a. Little interest or pleasure in doing things LittleInterest	1	2	3	4
b. Feeling down, depressed, or hopeless Hopeless	1	2	3	4
c. Feeling nervous, anxious, or on edge Nervous	1	2	3	4
d. Not being able to stop or control worrying Worrying	1	2	3	4

19011

© 2021 Williams CP et al. *JAMA Network Open.*
